# Effect of Exercise Training or Complex Mental and Social Activities on Cognitive Function in Adults With Chronic Stroke: A Randomized Clinical Trial

**DOI:** 10.1001/jamanetworkopen.2022.36510

**Published:** 2022-10-13

**Authors:** Teresa Liu-Ambrose, Ryan S. Falck, Elizabeth Dao, John R. Best, Jennifer C. Davis, Kim Bennett, Peter A. Hall, Ging-Yuek Robin Hsiung, Laura E. Middleton, Charles H. Goldsmith, Peter Graf, Janice J. Eng

**Affiliations:** 1 Department of Physical Therapy, University of British Columbia, Vancouver, British Columbia, Canada; 2 Djavad Mowafaghian Centre for Brain Health, University of British Columbia, Vancouver, British Columbia, Canada; 3 Centre for Hip Health and Mobility, Vancouver Coastal Health Research Institute, Vancouver, British Columbia, Canada; 4 School of Biomedical Engineering, University of British Columbia, Vancouver, British Columbia, Canada; 5 Gerontology Research Centre, Simon Fraser University, Vancouver, British Columbia, Canada; 6 Faculty of Management, University of British Columbia, Kelowna, British Columbia, Canada; 7 School of Public Health and Health Systems, University of Waterloo, Waterloo, Ontario, Canada; 8 Division of Neurology, University of British Columbia, Vancouver, British Columbia, Canada; 9 Clinic for Alzheimer Disease and Related Disorders, University of British Columbia, Vancouver, British Columbia, Canada; 10 Department of Kinesiology and Health Sciences, University of Waterloo, Waterloo, Ontario, Canada; 11 Faculty of Health Sciences, Simon Fraser University, Burnaby, British Columbia, Canada; 12 Department of Occupational Science and Occupational Therapy, University of British Columbia, Vancouver, British Columbia, Canada; 13 Department of Psychology, University of British Columbia, Vancouver, British Columbia, Canada; 14 Rehabilitation Research Program, GF Strong Rehabilitation Centre, Vancouver Coastal Health Research Institute, Vancouver, Canada

## Abstract

**Importance:**

A stroke doubles one’s risk for dementia. How to promote cognitive function among persons with chronic stroke is unclear.

**Objective:**

To evaluate the effect of exercise (EX) or cognitive and social enrichment activities (ENRICH) on cognitive function in adults with chronic stroke.

**Design, Setting, and Participants:**

This was a 3-group parallel, single-blinded, single-site, proof-of-concept randomized clinical trial at a research center in Vancouver, British Columbia, Canada. Participants included community-dwelling adults with chronic stroke, aged 55 years and older, able to walk 6 meters, and without dementia. The trial included a 6-month intervention and a 6-month follow-up. Randomization occurred from June 6, 2014, to February 26, 2019. Measurement occurred at baseline, 6 months, and 12 months. Data were analyzed from January to November 2021.

**Interventions:**

Participants were randomly allocated to twice-weekly supervised classes of: (1) EX, a multicomponent exercise program; (2) ENRICH, a program of cognitive and social enrichment activities; or (3) balance and tone (BAT), a control group that included stretches and light-intensity exercises.

**Main Outcomes and Measures:**

The primary outcome was the Alzheimer Disease Assessment Scale-Cognitive-Plus (ADAS-Cog-Plus), which included the 13-item ADAS-Cog, Trail Making Test Parts A and B, Digit Span Forward and Backward, Animal Fluency, and Vegetable Fluency.

**Results:**

One-hundred and twenty participants, with a mean (range) of 1.2 (1-4) strokes, a mean (SD) of 66.5 (53.8) months since the most recent stroke, mean (SD) baseline age of 70 (8) years, mean (SD) baseline ADAS-Cog-Plus of 0.22 (0.81), and 74 (62%) male participants, were randomized to EX (34 participants), ENRICH (34 participants), or BAT (52 participants). Seventeen withdrew during the 6-month intervention and another 7 during the 6-month follow-up. Including all 120 participants, at the end of the 6-month intervention, EX significantly improved ADAS-Cog-Plus performance compared with BAT (estimated mean difference: −0.24; 95% CI, −0.43 to −0.04; *P* = .02). This difference did not persist at the 6-month follow-up (estimated mean difference: −0.08; 95% CI, −0.29 to 0.12; *P* = .43). For the 13-item ADAS-Cog, the EX group improved by 5.65 points over the 6-month intervention (95% CI, 2.74 to 8.57 points; *P* < .001), exceeding the minimally clinical difference of 3.0 points.

**Conclusions and Relevance:**

These findings suggest that exercise can induce clinically important improvements in cognitive function in adults with chronic stroke. Future studies need to replicate current findings and to understand training parameters, moderators, and mediators to maximize benefits.

**Trial Registration:**

ClinicalTrials.gov identifier: NCT01916486

## Key Points

**Question:**

What is the effect of exercise or cognitive and social enrichment activities on cognitive function in adults with chronic stroke?

**Findings:**

In this randomized clinical trial with 120 participants, compared with control, exercise significantly improved cognitive function. The degree of improvement observed in the exercise group was clinically meaningful.

**Meaning:**

These findings suggest that exercise can induce clinically important improvements in cognitive function in adults with chronic stroke, a clinical population at increased risk for dementia.

## Teaser

This randomized clinical trial evaluates the effect of exercise training or complex mental and social activities to promote cognitive function in adults with chronic stroke.

## Introduction

A stroke doubles one’s risk for dementia.^1^ Stroke-related cognitive deficits are associated with reduced functional independence, institutionalization, reduced quality of life, and death.^2^ Thus, stroke survivors need interventions to promote cognitive function and to prevent dementia.

Exercise promotes cognitive and brain outcomes in older adults.^3,4^ However, there is insufficient high quality evidence for exercise as an efficacious intervention for cognitive function in stroke survivors.^5^ Notably, no published exercise trial among adults with chronic stroke was designed with cognitive function as the primary outcome.^5,6,7^

Environmental enrichment is defined as an intervention designed to increase motor, sensory, cognitive, and social activity by provision of a stimulating environment.^8^ The application of enrichment principles to promote cognitive health in stroke survivors is supported by evidence from animal^8^ and human studies.^9^ This 6-month, single-blinded, proof-of-concept randomized clinical trial assessed whether exercise or cognitive and social enrichment activities promote cognitive function in community-dwelling adults with chronic stroke.

## Methods

### Study Design and Setting

This was a 3-group parallel, single-blinded, single-site, proof-of-concept randomized clinical trial conducted in a research center (Vancouver, British Columbia, Canada). This study included a 6-month intervention and a 6-month follow-up (Figure). Ethical approval was provided by the University of British Columbia’s clinical research ethics board and the Vancouver Coastal Health Research Institute. All participants provided written informed consent in person. The trial protocol is published^10^ and presented in Supplement 1. This study followed the Consolidated Standards of Reporting Trials (CONSORT) reporting guideline.

**Figure.**
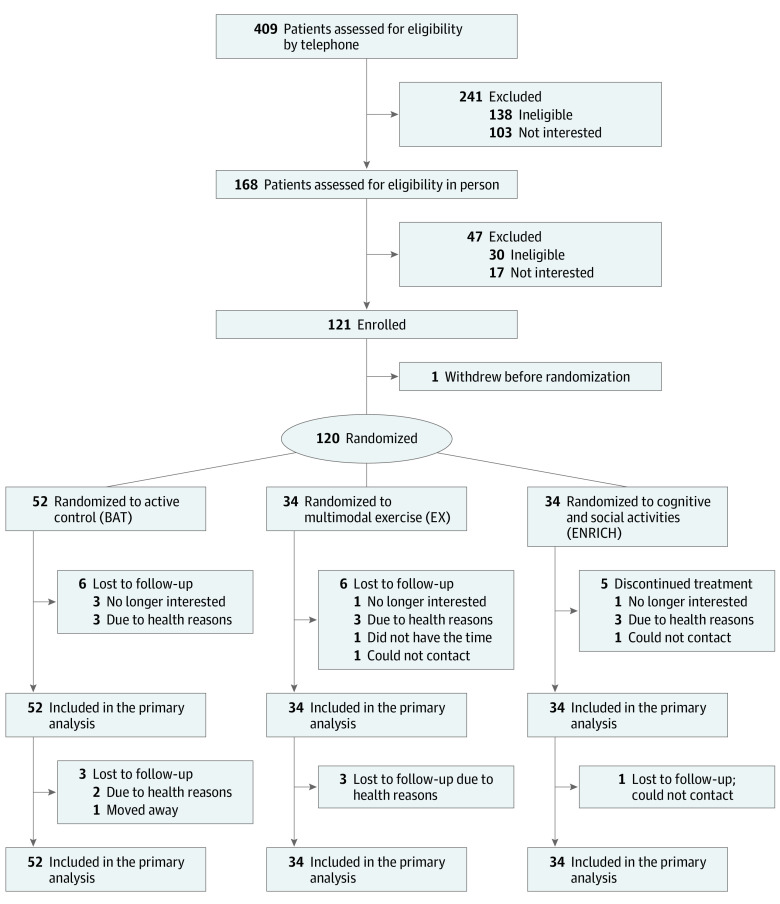
Trial Flow Diagram

### Recruitment

Participants were recruited from the community and stroke clinics. Enrollment and randomization occurred from June 6, 2014, to February 26, 2019.

### Inclusion and Exclusion Criteria

Eligible participants were community-dwelling adults who had an ischemic or hemorrhagic stroke. Additional inclusion criteria were: (1) aged 55 years or older; (2) had 1 or more strokes at least 12 months before study enrolment; (3) a Mini-Mental State Examination (MMSE)^11^ score of 20 of 30 or greater at screening, including a perfect score on the 3-step command to ensure intact comprehension and ability to follow instructions; (4) English-speaking; (5) not expected to start or were stable on a fixed dose of cognitive medications during the 12-month study period; (6) able to walk 6 meters with rest intervals with or without assistive devices; and (7) not currently participating in any regular therapy or progressive exercise. Exclusion criteria were neurodegenerative disease, dementia, at high risk for cardiac complications during exercise, taking medications that may negatively affect cognitive function, or aphasia as judged by an inability to communicate by telephone.

### Randomization and Blinding

The randomization sequence was generated by C.H.G. Participants were stratified by stroke status (1 vs ≥2 stroke events) and randomly allocated by an investigator (C.H.G.) not involved in running the trial to (1) multicomponent exercise training (EX); (2) cognitive and social enrichment activities (ENRICH); or (3) a control group consisting of stretching and toning exercises (balance and tone, or BAT) with an allocation ratio of 2:2:3 (EX:ENRICH:BAT, respectively) using permuted blocks within each stratum. The allocation ratio accounts for the 2 planned contrasts using the Dunnett Test^12^ (see Statistical Analysis section). Allocation was concealed.

Assessors were blinded to participants’ allocation and participants were asked to refrain from discussing their study involvement or experience during assessments with assessors. Participants and those who delivered the interventions were not blinded.

### Interventions

Each treatment group included twice-weekly supervised classes of 60 minutes for 6 months.^10^ All instructors were trained by the research team and delivered the interventions based on protocols. To minimize contamination, only one class occurred at any given time and there was a minimum of 30 minutes between classes. To ensure the physical safety of participants, the EX and BAT protocols included modifications.

The EX program was a multicomponent, progressive exercise intervention based on the Fitness and Mobility Exercise (FAME^13^) program.^14^ It included strength, aerobic, agility, and balance training exercises. Intensity of aerobic training was monitored by the Borg Rating of Perceived Exertion (RPE) and heart rate monitors (Polar RS400). Participants were asked initially to complete the EX sessions at an RPE of 12 (fairly light to somewhat difficult) and to progress to a maximum target RPE of 16 (difficult to very difficult).

The cognitive and social enrichment program was designed based on a prior pilot study^7^ and current evidence.^15^ It included computerized cognitive training^16^ and social and cognitive enrichment activities. Some activities used apps, and others were based on improvisation and mental activities from the Perk Activities program.^17^

The BAT program consisted of stretches, deep breathing and relaxation exercises, general posture education, grip strength and dexterity exercises, and light isometric toning exercises.^18^ Basic, nonprogressive exercises from the EX program were also included (eg, double calf raises, heel and toe tapping, tandem stand balance). Once a month, an education seminar replaced an exercise session.

### Measures

Measures were acquired at baseline, 6 months (ie, end of intervention), and 12 months (ie, 6-month follow-up) by blinded assessors. Adherence to interventions and current physical activity levels were assessed by unblinded research assistants.

The Functional Comorbidity Index^19^ measured the number of comorbid conditions. Global cognitive function was assessed by the MMSE^11^ and the Montreal Cognitive Assessment.^20^ The Center for Epidemiologic Studies Depression Scale assessed for depression.^21^ Motor function of the upper and lower extremities was assessed by the Fugl-Meyer Assessment Motor scale.^22^

### Outcomes

The Alzheimer Disease Assessment Scale-Cognitive-Plus (ADAS-Cog-Plus) was the primary outcome.^23^ The ADAS-Cog-Plus uses a multidimensional item response theory model to generate a global cognitive score from the 13-item ADAS-Cog^24^ and additional standard cognitive assessments. We used the 13-item ADAS-Cog, Trail Making Test Parts A and B, Digit Span Forward and Backward, Animal Fluency, and Vegetable Fluency as the input variables into the scoring algorithm. These tests collectively assess cognitive domains commonly impacted by stroke—memory, attention, and executive functions. The range of ADAS-Cog-Plus scores is from −3.46 to 4.31; this is on a scale with a mean of 0 and a SD of 1. Scores of approximately −1.0 indicate healthy cognitive functioning, scores of 0.0 indicate mild cognitive impairment, and scores of 1.0 indicate dementia.^23^ For the 13-item ADAS-Cog, a change of 3.0 points is a minimally important difference.^25^

Secondary measures were a computerized version of the Stroop task, self-report Lawton and Brody Instrumental Activities of Daily Living (IADL) Scale,^26^ Short Physical Performance Battery (SPPB),^27^ 4-meter gait speed, and 6-Minute Walk Test (6MWT).^28^ For stroke survivors, small meaningful change in the SPPB is 0.5 points; in gait speed, 0.05 m/s; and in 6MWT, 20 meters; substantial change in the SPPB is 1.0 points; in gait speed, 0.10 m/s; and in 6MWT, 50 meters.^29^ Current physical activity level was determined by the Community Health Activities Model Program for Seniors (CHAMPS) questionnaire.^30^

### Adverse Events and Adherence

During the 6-month intervention period, participants were instructed to report any adverse events. Adherence was determined from class attendance and calculated as the percentage of total classes attended.

### Statistical Analysis

All analyses were conducted in R version 3.5.1 (R Project for Statistical Computing).^31^ This trial allows the evaluation of statistical significance of the treatment effect between groups on the ADAS-Cog-Plus at the end of the 6-month intervention.^10^ According to our published work,^32^ we first assumed a standardized effect size of 0.6 for exercise on cognitive function, as measured by the 11-item ADAS-Cog. With an α of .05, 39 participants per group would provide a power greater than 0.80. We then assumed a standardized effect size of 0.7 for the ADAS-Cog-Plus, as it has greater sensitivity to changes in cognitive function compared with the 11- or 13-item ADAS-Cog.^23^ After accounting for 15% attrition and 2:2:3 allocation, 34 participants were randomized to the EX group, 34 to the ENRICH group, and 52 to the BAT group, for a total sample of 120.

Treatment effects for our primary outcome, ADAS-Cog-Plus, were evaluated using linear mixed models with restricted maximum likelihood estimation using the lme4 version 1.1-27.1 and lsmeans version 2.30-0 packages. The model included random intercepts, and fixed effects of time at baseline, 6 months, and 12 months, group assignment (ie, EX, ENRICH, BAT) and their interaction. Unequal variance was allowed across time and group.

We performed 2 planned contrasts using the Dunnett Test,^12^ a multiple comparison procedure, to assess differences in ADAS-Cog-Plus at 6 months between: (1) EX vs BAT; and (2) ENRICH vs BAT. The overall α was set at .05 (2-sided). Baseline ADAS-Cog-Plus and MMSE scores were included as fixed-effect covariates. A complete case analysis was also conducted, in which participants with valid data at all time points were included (eTable 1 in Supplement 2). To facilitate the interpretation of treatment effects on cognitive function, we conducted a post hoc analysis of the 13-item ADAS-Cog using the same linear mixed model, with baseline 13-item ADAS-Cog and MMSE scores as fixed-effect covariates.

Linear mixed models with restricted maximum likelihood estimation were also conducted on the following secondary outcomes: Stroop task, 4-meter gait speed, 6MWT, and CHAMPS. For models examining treatment effects on 4-meter gait speed, 6MWT, and CHAMPS, baseline value of outcome and baseline Fugl-Meyer Assessment Motor score were included as fixed-effect covariates. For the Stroop task, baseline value and baseline MMSE score were included as fixed-effect covariates. Estimated marginal means, within group differences from baseline, and between group differences for planned contrasts at 6 months and at 12 months were calculated for all models.

We conducted negative binomial generalized linear mixed models in the lme4 package for the secondary outcomes of IADL and SPPB because of their nonnormal distribution. Baseline value of outcome and baseline Fugl-Meyer Assessment Motor score were included as fixed-effect covariates. We used the package ggeffects version 1.1.2 to determine estimates of marginal means, within group differences from baseline, and between group differences for planned contrasts at 6 months and 12 months.

Tertiary analyses were conducted to assess whether changes in ADAS-Cog-Plus correlated with changes in IADL and the SPPB over the 6-month intervention. We calculated the Spearman ρ for these analyses. Data were analyzed from January to November 2021.

## Results

One-hundred and twenty participants were enrolled, 34 participants were randomized to the EX group, 34 to the ENRICH group, and 52 to the BAT group; their mean (SD) baseline age was 70 (8) years and 74 (62%) were male participants (Table 1). The mean (SD) baseline Montreal Cognitive Assessment and ADAS-Cog-Plus scores of 21.89 (4.15) and 0.22 (0.81), respectively, indicate participants had cognitive impairment.^20,23^ The mean (SD) baseline Fugl-Meyer Assessment Motor score of 81.21 (23.85) indicates moderate to mild motor impairment.^33^

**Table 1.** Sample Characteristics by Treatment Group

Variable	Participants, Mean (SD)		
	EX (n = 34)	ENRICH (n = 34)	BAT (n = 52)
Age, y	70.65 (9.14)	71.29 (9.25)	70.42 (7.81)
Sex, No. (%)			
Male	21 (62)	23 (68)	30 (58)
Female	13 (38)	11 (32)	22 (42)
Height, cm	166.31 (10.51)	170.2 (9.59)	166.21 (9.25)
Weight, kg	75.94 (13.22)	79.95 (17.99)	77.96 (16.06)
Body mass index^a^	27.37 (3.69)	27.32 (4.45)	28.11 (4.89)
Level of education, No. (%)			
High school diploma or less	6 (18)	5 (15)	13 (25)
Some college or university	10 (29)	8 (23)	18 (35)
University degree or higher	18 (53)	21 (62)	21 (40)
No. of strokes	1.09 (0.29)	1.15 (0.50)	1.29 (0.72)
Time since most recent stroke, mo	67.53 (48.27)	62.00 (55.60)	72.29 (57.04)
Type of stroke, No. (%)			
Hemorrhagic	8 (23)	10 (29)	15 (29)
Ischemic	22 (65)	18 (53)	33 (63)
Lacunar	3 (9)	3 (9)	1 (2)
Ischemic and Lacunar	0	0	1 (2)
Unknown	1 (3)	3 (9)	2 (4)
Functional Comorbidity Index (0-18)^b^	3.44 (2.05)	2.97 (1.49)	3.67 (1.78)
Instrumental Activities of Daily Living (0-8)^c^	6.82 (1.83)	7.00 (1.26)	6.79 (1.73)
Mini-Mental State Examination (0-30)^d^	27.15 (2.56)	27.56 (2.52)	27.15 (2.40)
Montreal Cognitive Assessment (0-30)^e^	21.26 (3.70)	22.82 (3.99)	21.69 (4.49)
Center for Epidemiological Studies-Depression Scale (0-60)^f^	8.74 (6.31)	7.88 (9.45)	10.70 (8.47)
Fugl-Meyer Assessment Motor Score (0-100)^g^	73.25 (26.48)	80.09 (24.57)	87.38 (19.91)
Alzheimer Disease Assessment Scale-Cognitive-Plus^h^	0.39 (0.77)	0.12 (0.71)	0.16 (0.88)
13-item Alzheimer Disease Assessment Scale-Cognitive (0-90)^i^	18.16 (7.47)	16.42 (6.29)	17.19 (8.00)
Stroop Interference Ratio^j^	0.16 (0.11)	0.16 (0.12)	0.16 (0.15)
Short Physical Performance Battery (0-12)^k^	7.44 (2.52)	8.15 (2.48)	8.69 (2.81)
Gait speed, m/s	0.76 (0.32)	0.88 (0.35)	0.91 (0.31)
Six-minute walk test, m	291.09 (147.83)	342.47 (143.44)	340.79 (131.85)
Physical activity, kcal/wk^l^	3262.16 (2499.61)	3535.41 (2717.95)	3536.30 (2255.00)

^a^ Body mass index is calculated as weight in kilograms divided by height in meters squared.

^b^ The Functional Comorbidity Index ranges from 0 (no comorbid illness) to 18 (highest number of comorbid illness). Higher scores are associated with lower physical function.^19^

^c^ The (Lawton) Instrumental Activities of Daily Living ranges from 0 (dependent) to 8 (independent). A score of 7 would indicate someone who is largely independent but cannot manage finances or perform housekeeping tasks.

^d^ The Mini-Mental State Examination ranges from 0 (lowest) to 30 (highest); scores 24 to 30 are the reference range.

^e^ The Montreal Cognitive Assessment ranges from 0 (lowest) to 30 (highest); scores 26 to 30 are the reference range.

^f^ The 20-item Centre for Epidemiological Studies-Depression Scale ranges from 0 (no depressive symptoms) to 60 (severe depressive symptoms).

^g^ The Fugl-Meyer Assessment Motor Score ranges from 0 (no impairment) to 100; scores greater than 79 are indicative of mild motor impairment, 56 to 79 indicate moderate impairment, 36 to 55 indicate severe impairment, and 0 to 35 indicate very severe impairment.

^h^ Lower Alzheimer Disease Assessment Scale-Cognitive-Plus scores indicate better cognitive performance. The range of ADAS-Cog-Plus scores is from −3.46 to 4.31. Scores approximately of −1.0 indicate healthy cognitive functioning, of 0.0 indicate mild cognitive impairment, and of 1.0 indicate dementia.

^i^ Lower 13-item Alzheimer Disease Assessment Scale-Cognitive scores indicate better cognitive performance.

^j^ Stroop Interference Ratio is calculated as: incongruent median reaction time (in milliseconds) − congruent median reaction time (in milliseconds) / congruent median reaction time (in milliseconds); lower scores indicate less interference, or better performance.

^k^ The Short Physical Performance Battery ranges from 0 to 12; scores of 9 or lower indicate increased risk for disability.

^l^ Measured using the Community Health Model Activities Program physical activity questionnaire.

The final measurements were made on March 3, 2020, and were not impacted by the COVID-19 pandemic. We screened 409 individuals by telephone, which led to in-person screening of 168 individuals (Figure). Seventeen individuals declined to participate, 30 were ineligible, and 1 withdrew before randomization. The observed recruitment rate was 50% (ie, 120 of 241). The attrition rate during the 6-month intervention was 14% (ie, 17 of 120). Seven additional participants withdrew during the 6-month follow-up (ie, 20% attrition) (Figure).

### Primary Outcome

At the end of the 6-month intervention, participants in the EX group had significantly better ADAS-Cog-Plus performance compared with the BAT group (estimated mean difference: −0.24; 95% CI, −0.43 to −0.04; *P* = .02; Table 2). This difference did not persist at the end of the 6-month follow-up (estimated mean difference: −0.08; 95% CI, −0.29 to 0.12; Table 3). There were no differences in ADAS-Cog-Plus performance between the ENRICH group and the BAT group at the end of the intervention (estimated mean difference: −0.11; 95% CI, −0.30 to 0.09) or at the end of the 6-month follow-up (estimated mean difference: −0.08, 95% CI: −0.28 to 0.12). The results of the complete case analysis of ADAS-Cog-Plus are in eTable 1 in Supplement 2.

**Table 2.** Estimated Within-Group Change and Between-Group Difference at End of Intervention in Primary and Secondary Outcome Variables

Variable	Adjusted within-group change from baseline to month 6 (SE)^a^			Adjusted between-group difference at month 6 (95% CI)			
	EX (n = 34)	ENRICH (n = 34)	BAT (n = 52)	EX vs BAT	*P* value	ENRICH vs BAT	*P* value
Primary outcome: Alzheimer Disease Assessment Scale-Cognitive-Plus	0.71 (0.15)^b^	0.31 (0.14)^b^	0.24 (0.14)	−0.24 (−0.43 to −0.04)	.02	−0.11 (−0.30 to 0.09)	.27
Secondary outcomes							
13-item Alzheimer Disease Assessment Scale-Cognitive	5.65 (1.49)^b^	2.43 (1.32)	2.24 (1.27)	−2.44 (−4.36 to −0.52)	.01	−0.96 (−2.87 to 0.96)	.32
Stroop Interference Ratio^c^	0.01 (0.03)	0.03 (0.03)	0.01 (0.02)	0.00 (−0.05 to 0.04)	.84	−0.02 (−0.07 to 0.02)	.32
Instrumental Activities of Daily Living	−0.62 (1.08)	−0.42 (1.06)	−0.87 (1.07)	−0.22 (−3.11 to 2.66)	.56	−0.25 (−3.13 to 2.64)	.57
Short Physical Performance Battery	−1.81 (1.09)	−0.99 (1.09)	−1.07 (1.08)	−0.51(−3.30 to 2.27)	.64	−0.62 (−3.41 to 2.16)	.67
Gait speed, m/s	−0.16 (0.06)^b^	−0.04 (0.07)	−0.05 (0.05)	−0.04 (−0.12 to 0.05)	.37	−0.04 (−0.13 to 0.04)	.04
Six-minute walk test, m	−68.72 (27.20)^b^	−0.15 (26.44)	−27.53 (19.97)	−8.51 (−34.00 to 16.94)	.51	−25.70 (−50.70 to −0.73)	.31
Physical activity, kcal/wk^d^	369 (549)	299 (569)	−392 (419)	−1035 (−1912 to −157)	.02	−692 (−1539 to 155)	.11

^a^ Change calculated as baseline score minus month 6 score. Positive within-group change scores for Alzheimer Disease Assessment Scale-Cognitive-Plus and 13-item Alzheimer Disease Assessment Scale indicate improvement. Negative within-group change scores for Stroop Interference Ratio, Lawson Instrumental Activities of Daily Living, Short Physical Performance Battery, 6-Minute Walk Test, Gait Speed, and Physical Activity indicate improvement. Within-group change scores are adjusted for baseline Mini-Mental State Examination.

^b^ Significant (*P* < .05) within-group change from baseline to month 6.

^c^ Stroop Interference Ratio is calculated as: incongruent median reaction time (in milliseconds) − congruent median reaction time (in milliseconds) / (in milliseconds).

^d^ Measured using the Community Health Model Activities Program physical activity questionnaire.

**Table 3.** Estimated Within-Group Change and Between-Group Difference at End of Follow-up in Primary and Secondary Outcome Variables

Variable	Adjusted within-group change from baseline to month 12 (SE)^a^			Adjusted between-group difference at month 12 (95% CI)			
	EX (n = 34)	ENRICH (n = 34)	BAT (n = 52)	EX vs BAT	*P* value	ENRICH vs BAT	*P* value
Primary outcome: Alzheimer Disease Assessment Scale-Cognitive-Plus	0.61 (0.15)^b^	0.33 (0.14)^b^	0.29 (0.14)^b^	−0.08 (−0.29 to 0.12)	.43	−0.08 (−0.28 to 0.12)	.44
Secondary outcomes							
13-item Alzheimer Disease Assessment Scale-Cognitive	5.19 (1.50)^b^	2.98 (1.32)^b^	3.84 (1.27)^b^	−0.37 (−2.36 to 1.61)	.71	0.09 (−1.85 to 2.04)	.93
Stroop Interference Ratio^c^	0.02 (0.03)	0.01 (0.03)	0.00 (0.02)	−0.02 (−0.06 to 0.03)	.49	−0.01 (−0.06 to 0.04)	.64
Instrumental Activities of Daily Living	−0.74 (1.09)	−0.63 (1.07)	−0.93 (1.07)	−0.17 (−3.11 to 2.74)	.55	−0.09 (−2.99 to 2.82)	.55
Short Physical Performance Battery	−1.86 (1.09)	−0.72 (1.09)	−0.39 (1.08)	0.23 (−3.30 to 3.01)	.87	−0.21 (−2.99 to 2.58)	.61
Gait speed, m/s	−0.12 (0.06)^b^	−0.01 (0.07)	0.01 (−0.05)	−0.02 (−0.11 to 0.07)	.62	−0.01 (−0.09 to 0.07)	.82
Six-minute walk test, m	−50.42 (27.30)	6.35 (26.48)	−8.64 (20.03)	−7.92 (−34.00 to 18.11)	.55	−13.32 (−38.60 to 11.99)	.30
Physical activity, kcal/wk^d^	596 (555)	489 (581)	−257 (432)	−1127 (−2048 to −206)	.02	−747 (−1649 to 156)	.10

^a^ Change calculated as baseline score minus month 12 score. Positive within-group change scores for Alzheimer Disease Assessment Scale-Cognitive-Plus and the 13-item indicate Alzheimer Disease Assessment Scale-Cognitive improvement. Negative within group change scores for Stroop Interference Ratio, Lawson Instrumental Activities of Daily Living, Short Physical Performance Battery, 6-minute walk test, gait speed, and physical activity indicate improvement. Within group change scores are adjusted for baseline Mini-Mental State Examination.

^b^ Significant (*P* < .05) within-group change from baseline to month 12.

^c^ Stroop Interference Ratio is calculated as: incongruent median reaction time (in milliseconds) − congruent median reaction time (in milliseconds) / congruent median reaction time (in milliseconds).

^d^ Measured using the Community Health Model Activities Program physical activity questionnaire.

### Secondary Outcomes

At the end of the 6-month intervention, the BAT group performed significantly better on the 6MWT compared with the ENRICH group (estimated mean difference: −25.70 meters; 95% CI, −50.70 to −0.73 meters; *P* = .04). Participants of the BAT group also reported significantly higher physical activity participation outside the research study than the EX group at the end of the 6-month intervention (estimated mean difference: −1035 kcal/wk, 95% CI: −1912 to −157 kcal/wk; *P* = .02), and at the end of the 6-month follow-up (estimated mean difference: −1127 kcal/wk; 95% CI, −2048 to −206 kcal/wk; *P* = 0.02). There were no other between-group differences in secondary outcome measures at the end of the 6-month intervention (Table 2) or at the end of the 6-month follow-up (Table 3). However, it is noteworthy that the EX group significantly and meaningfully improved on the 4-meter gait speed and the 6MWT over the 6-month intervention.^29^ Specifically, the 4-meter gait speed improved by 0.16 m/s and the 6MWT improved by 68.72 m (Table 2).

### Tertiary Outcomes

Changes in ADAS-Cog-Plus score were not associated with changes in IADL (ρ = −0.15; *P* = .13). Changes in ADAS-Cog-Plus score were also not associated with changes in the SPPB (ρ = 0.02; *P* = .85).

### Post Hoc Analysis

At the end of the 6-month intervention, participants in the EX group had significantly better 13-item ADAS-Cog performance compared with the BAT group (estimated mean difference: −2.44; 95% CI, −4.36 to −0.52; *P* = .01) (Table 2). This difference did not persist at the end of the 6-month follow-up (estimated mean difference: −0.37; 95% CI, −2.36 to 1.61; Table 3). There were no differences between the ENRICH group and the BAT group at the end of the intervention (estimated mean difference: −0.96; 95% CI to −2.87 to 0.96) or at the end of the 6-month follow-up (estimated mean difference: 0.09; 95% CI, −1.85 to 2.04). Participants in the EX group improved on the 13-item ADAS-Cog by 5.65 points (95% CI, 2.74 to 8.57 points; *P* < .001) (Table 2), exceeding the established minimally clinically important difference of 3.0 points.^25^

### Adverse Events and Adherence

A total of 42 adverse events were reported, 19 in the EX group, 2 in the ENRICH group, and 21 in the BAT group. Thirty-five events were deemed to be not related or most likely not related to the study. Of the remaining 7 possibly or definitely related adverse events, 4 were in the EX group and 3 were in the BAT group. For the EX group, all 4 events resolved without additional medical attention or intervention. For the BAT group, 2 events resolved without additional medical attention or intervention. One event was slight numbness and marked fatigue on the stroke-affected side that resolved after 4 days. The participant was seen by their physician with no subsequent medical testing or intervention recommended. Mean adherence was 80% (41.6 of 52 sessions) for the EX group, 82% (40.6 of 52 sessions) for the ENRICH group, and 78% (42.6 of 52 sessions) for the BAT group. For the EX group, the mean (SD) RPE was 12 (1.8) (ie, “light” to “somewhat hard”) in month 1 and in month 6, it was 14 (1.7) (ie, “somewhat hard” to “hard”).

## Discussion

Multicomponent exercises induced clinically important improvements in cognitive function in adults with chronic stroke. Stretching and toning exercises improved 6MWT compared with cognitive and social enrichment activities.

This 6-month trial with follow-up of 120 participants with chronic stroke is the longest we know of with cognitive function as the primary outcome.^5^ The observed benefit of multicomponent exercises on ADAS-Cog-Plus concurs with prior findings from a meta-analysis that found combined aerobic and resistance training programs generated the largest cognitive gains, and improved cognitive function can be observed in the chronic stroke phase (mean 2.6 years after stroke).^5^ Our study extends prior meta-analytic findings by demonstrating exercise can improve cognitive domains impacted by stroke—memory, attention, and executive functions. The meta-analysis found a significant effect of exercise on attention, but not on memory and executive functions.^5^ The version of ADAS-Cog-Plus used in this study assessed memory, attention, and executive functions.

Notably, the primary and post hoc results show exercise induced a shift from the state of cognitive impairment to more normal cognitive functioning and the degree of improvement is clinically important. Specifically, participants randomized to the multicomponent exercise intervention had a mean baseline ADAS-Cog-Plus score of 0.39. At the end of the 6-month intervention period, their mean ADAS-Cog-Plus score was −0.32 (eTable 2 in Supplement 2). The post hoc results show participants in the exercise group improved on the 13-item ADAS-Cog by 5.65 points (Table 2), exceeding the established minimally clinically important difference of 3.0 points.^25^

Cognitive and social enrichment activities did not improve cognitive function and this aligns with prior findings. A 2 × 2 factorial design 10-week trial examined the independent and synergistic effects of aerobic exercise and computerized cognitive training on fluid intelligence in 52 adults who were more than 6 months poststroke and found no significant benefit of computerized cognitive training alone compared with control.^34^

Stretching and toning exercises improved 6MWT compared with cognitive and social enrichment activities. The degree of improvement in 6MWT was small but meaningful (ie, ≥20 meters).^29^ It is plausible the few basic FAME exercises delivered to those in the control group improved 6MWT by inducing a small meaningful change in gait speed of 0.05 m/s (Table 2).^29^

The control group also reported significantly higher physical activity participation compared with the exercise group. Participants in the exercise group may have engaged in less structured physical activity outside of the research study because they were receiving a moderate-intensity multicomponent exercise program. However, this difference was still evident at the end of the 6-month follow-up. This finding highlights the need to embed health behavior change strategies (eg, brief action planning) with structured exercise interventions to substantially impact long-term health.

### Limitations

This trial has several limitations. First, in terms of lesion type and location, this study sample of adults with chronic stroke is a heterogeneous sample and this may have limited our ability to detect between-group differences. Thus, we may be providing conservative estimates of efficacy of multicomponent exercise training on cognitive function in adults with chronic stroke. Second, the study sample included chronic stroke survivors with mild to moderate motor impairment and thus, limits the generalizability of results to those with more severe stroke-related motor impairments. Third, due to the diverse content of the cognitive and social enrichment intervention, there may have been insufficient dose and specificity of training for the cognitive domains of memory, attention, and executive functions measured by the ADAS-Cog-Plus. Additionally, we were likely underpowered for the 6-month follow-up analysis as there was 20% attrition.

## Conclusions

The findings of this randomized clinical trial suggest that exercise can induce clinically important improvements in cognitive function in adults with chronic stroke and, thus, should be recommended to promote cognitive health in this clinical population at risk for dementia. The exercise intervention (FAME) has already been implemented worldwide, making it a turnkey strategy to reshape the path of cognitive aging among community-dwelling adults with chronic stroke. Future studies need to replicate current findings and to understand training parameters, moderators, and mediators to maximize benefits.

## Notes

Supplement 1.
Trial Protocol

Supplement 2. **eTable 1.** Estimated Between-Group Difference at Month 6 and Month 12 for Complete Case Analyses **eTable 2.** Estimated Marginal Means for Primary and Secondary Outcome Variables by Treatment Group

Supplement 3.
Data Sharing Statement

## References

[1] Kokmen E, Whisnant JP, O’Fallon WM, Chu CP, Beard CM. Dementia after ischemic stroke: a population-based study in Rochester, Minnesota (1960-1984). Neurology. 1996;46(1):154-159. doi:10.1212/WNL.46.1.154 8559366

[2] Donkor ES. Stroke in the 21(st) century: a snapshot of the burden, epidemiology, and quality of life. Stroke Res Treat. 2018:3238165. doi:10.1155/2018/3238165 30598741 PMC6288566

[3] Northey JM, Cherbuin N, Pumpa KL, Smee DJ, Rattray B. Exercise interventions for cognitive function in adults older than 50: a systematic review with meta-analysis. Br J Sports Med. 2018;52(3):154-160. doi:10.1136/bjsports-2016-096587 28438770

[4] Intzandt B, Vrinceanu T, Huck J, . Comparing the effect of cognitive vs. exercise training on brain MRI outcomes in healthy older adults: a systematic review. Neurosci Biobehav Rev. 2021;128:511-533. doi:10.1016/j.neubiorev.2021.07.003 34245760

[5] Oberlin LE, Waiwood AM, Cumming TB, Marsland AL, Bernhardt J, Erickson KI. Effects of physical activity on poststroke cognitive function: a meta-analysis of randomized controlled trials. Stroke. 2017;48(11):3093-3100. doi:10.1161/STROKEAHA.117.017319 28931620 PMC5784766

[6] Tang A, Eng JJ, Krassioukov AV, Tsang TS, Liu-Ambrose T. High- and low-intensity exercise do not improve cognitive function after stroke: a randomized controlled trial. J Rehabil Med. 2016;48(10):841-846. doi:10.2340/16501977-2163 27786346

[7] Liu-Ambrose T, Eng JJ. Exercise training and recreational activities to promote executive functions in chronic stroke: a proof-of-concept study. J Stroke Cerebrovasc Dis. 2015;24(1):130-137. doi:10.1016/j.jstrokecerebrovasdis.2014.08.012 25440324 PMC4486380

[8] Nithianantharajah J, Hannan AJ. Enriched environments, experience-dependent plasticity and disorders of the nervous system. Nat Rev Neurosci. 2006;7(9):697-709. doi:10.1038/nrn1970 16924259

[9] Valenzuela MJ, Sachdev P. Brain reserve and dementia: a systematic review. Psychol Med. 2006;36(4):441-454. doi:10.1017/S0033291705006264 16207391

[10] Best JR, Eng JJ, Davis JC, . Study protocol for Vitality: a proof-of-concept randomised controlled trial of exercise training or complex mental and social activities to promote cognition in adults with chronic stroke. BMJ Open. 2018;8(3):e021490. doi:10.1136/bmjopen-2018-021490 29550783 PMC5875626

[11] Folstein MF, Folstein SE, McHugh PR. “Mini-mental state”. A practical method for grading the cognitive state of patients for the clinician. J Psychiatr Res. 1975;12(3):189-198. doi:10.1016/0022-3956(75)90026-6 1202204

[12] Dunnett C, Goldsmith C. When and how to decide to do multiple comparisons. In: Buncher CR, Tsay J-Y, eds. Statistics in the pharmaceutical company. 3rd ed. CRC Press, Taylor and Francis Group; 2006:421-451.

[13] The Fitness and Mobility Exercise Program. FAME: Fitness and Mobility Exercise Program. Accessed August 31, 2022. https://fameexercise.com/

[14] Pang MY, Eng JJ, Dawson AS, McKay HA, Harris JE. A community-based fitness and mobility exercise program for older adults with chronic stroke: a randomized, controlled trial. J Am Geriatr Soc. 2005;53(10):1667-1674. doi:10.1111/j.1532-5415.2005.53521.x 16181164 PMC3226792

[15] Basak C, Boot WR, Voss MW, Kramer AF. Can training in a real-time strategy video game attenuate cognitive decline in older adults? Psychol Aging. 2008;23(4):765-777. doi:10.1037/a0013494 19140648 PMC4041116

[16] Hardy JL, Nelson RA, Thomason ME, . Enhancing cognitive abilities with comprehensive training: a large, online, randomized, active-controlled trial. PLoS One. 2015;10(9):e0134467. doi:10.1371/journal.pone.0134467 26333022 PMC4557999

[17] Moritz R, Neeson I, Latif F, Constantine B. Perk activities: the social experience that stimulates. 2017. Accessed December 6, 2017. https://www.perkactivities.com

[18] Liu-Ambrose T, Khan KM, Eng JJ, Janssen PA, Lord SR, McKay HA. Resistance and agility training reduce fall risk in women aged 75 to 85 with low bone mass: a 6-month randomized, controlled trial. J Am Geriatr Soc. 2004;52(5):657-665. doi:10.1111/j.1532-5415.2004.52200.x 15086643 PMC3344816

[19] Groll DL, To T, Bombardier C, Wright JG. The development of a comorbidity index with physical function as the outcome. J Clin Epidemiol. 2005;58(6):595-602. doi:10.1016/j.jclinepi.2004.10.018 15878473

[20] Nasreddine ZS, Phillips NA, Bédirian V, . The Montreal Cognitive Assessment, MoCA: a brief screening tool for mild cognitive impairment. J Am Geriatr Soc. 2005;53(4):695-699. doi:10.1111/j.1532-5415.2005.53221.x 15817019

[21] Radloff L. The CES-D scale: a self-reported depression scale for research in the general population. Appl Psychol Meas. 1977;1(3):385-401. doi:10.1177/014662167700100306

[22] Fugl-Meyer AR, Jääskö L, Leyman I, Olsson S, Steglind S. The post-stroke hemiplegic patient. 1. a method for evaluation of physical performance. Scand J Rehabil Med. 1975;7(1):13-31. 1135616

[23] Skinner J, Carvalho JO, Potter GG, ; Alzheimer’s Disease Neuroimaging Study. The Alzheimer's Disease Assessment Scale-Cognitive-Plus (ADAS-Cog-Plus): an expansion of the ADAS-Cog to improve responsiveness in MCI. Brain Imaging Behav. 2012;6(4):489-501. doi:10.1007/s11682-012-9166-3 22614326 PMC3873823

[24] Mohs RC, Knopman D, Petersen RC, . Development of cognitive instruments for use in clinical trials of antidementia drugs: additions to the Alzheimer’s Disease Assessment Scale that broaden its scope. The Alzheimer’s Disease Cooperative Study. Alzheimer Dis Assoc Disord. 1997;11(suppl 2):S13-S21. doi:10.1097/00002093-199700112-00003 9236948

[25] Schrag A, Schott JM, Alzheimer’s Disease Neuroimaging Initiative. What is the clinically relevant change on the ADAS-Cog? J Neurol Neurosurg Psychiatry. 2012;83(2):171-173. doi:10.1136/jnnp-2011-300881 22019547

[26] Lawton MP, Brody EM. Assessment of older people: self-maintaining and instrumental activities of daily living. Gerontologist. 1969;9(3):179-186. doi:10.1093/geront/9.3_Part_1.179 5349366

[27] Guralnik JM, Ferrucci L, Simonsick EM, Salive ME, Wallace RB. Lower-extremity function in persons over the age of 70 years as a predictor of subsequent disability. N Engl J Med. 1995;332(9):556-561. doi:10.1056/NEJM199503023320902 7838189 PMC9828188

[28] Enright PL, McBurnie MA, Bittner V, ; Cardiovascular Health Study. The 6-min walk test: a quick measure of functional status in elderly adults. Chest. 2003;123(2):387-398. doi:10.1378/chest.123.2.387 12576356

[29] Perera S, Mody SH, Woodman RC, Studenski SA. Meaningful change and responsiveness in common physical performance measures in older adults. J Am Geriatr Soc. 2006;54(5):743-749. doi:10.1111/j.1532-5415.2006.00701.x 16696738

[30] Stewart AL, Mills KM, King AC, Haskell WL, Gillis D, Ritter PL. CHAMPS physical activity questionnaire for older adults: outcomes for interventions. Med Sci Sports Exerc. 2001;33(7):1126-1141. doi:10.1097/00005768-200107000-00010 11445760

[31] Falck R. All analyses and output for the Vitality Study. August 13, 2022. Accessed September 7, 2022. https://github.com/ryanfalck/Vitality-Study

[32] Liu-Ambrose T, Best JR, Davis JC, . Aerobic exercise and vascular cognitive impairment: a randomized controlled trial. Neurology. 2016;87(20):2082-2090. doi:10.1212/WNL.0000000000003332 27760869 PMC5109938

[33] Duncan PW, Goldstein LB, Horner RD, Landsman PB, Samsa GP, Matchar DB. Similar motor recovery of upper and lower extremities after stroke. Stroke. 1994;25(6):1181-1188. doi:10.1161/01.STR.25.6.1181 8202977

[34] Ploughman M, Eskes GA, Kelly LP, . Synergistic benefits of combined aerobic and cognitive training on fluid intelligence and the role of IGF-1 in chronic stroke. Neurorehabil Neural Repair. 2019;33(3):199-212. doi:10.1177/1545968319832605 30816066

