# Supplementary material for: Effect of Exercise Training or Complex Mental and Social Activities on Cognitive Function in Adults With Chronic Stroke: A Randomized Clinical Trial
Source: JAMA Netw Open. 2022 Oct 13;5(10):e2236510. doi: 10.1001/jamanetworkopen.2022.36510 (PMC9561961; doi:10.1001/jamanetworkopen.2022.36510)
Supplement: Supplement 1. — Trial Protocol [file jamanetwopen-e2236510-s001.pdf]

Supplementary 1: Study protocol

Introduction

One in six older adults will suffer a stroke in their lifetime, or one stroke every two seconds worldwide [1]. Of relevance to our study, cerebrovascular disease – such as stroke – is the second most common cause of dementia (i.e., vascular dementia) [2-5], accounting for up to 38% of all dementia cases [6]. Specifically, a stroke doubles one's risk for dementia [7]. Moreover, impairments in several domains of cognition—including memory, attention, and executive function—are common following stroke [8-10]. Stroke-related cognitive deficits are associated with other negative outcomes including institutionalization [11], reduced quality of life [12], and death [13]. Thus, stroke survivors represent a target population in need of intervention strategies to promote cognitive function and prevent dementia. However, the current standard of care in stroke rehabilitation does not adequately address the clinically important cognitive consequences of stroke – especially for those who are in the chronic phase (i.e., > 12 months since an index stroke).

Current evidence from randomized controlled trials (RCTs) suggests that targeted exercise training is an effective strategy to promote both cognitive and functional brain plasticity in older adults [14-20]. A meta-analysis concluded that aerobic training has robust but selective benefits for cognitive function; the largest benefits occur for executive function [15]. Rodent models have shown that exercise training induces upregulation of neurotrophic factors within the central nervous system that, in turn, contribute to neural health [21 22] and myelin recovery following pathological insult [23 24].

However, there is insufficient quality evidence for targeted exercise training as an effective strategy to promote cognitive function in stroke survivors [25 26]. Despite the high prevalence of cognitive deficits and the increased risk for dementia in this population, few randomized controlled trials (RCTs) to date [27-29] have focused on targeted exercise training on cognitive function. A small-scale RCT of individuals with chronic stroke ($\geq$ 6 months post ischemic stroke) showed improved processing speed following 8 weeks of aerobic exercise training but no effects on other aspects of cognition compared to 8 weeks of stretching [29]. A second small-scale RCT found that a 19-week multi-component exercise training program (adapted from the Fitness and Mobility Exercise [FAME] program) improved general cognition and increased cerebral blood flow compared to a strength program of equal length [30]. A pre-post designed study showed that general cognition and executive function were improved following 6 months of combined aerobic and resistance training in individuals who had experienced a stroke at least 10 weeks prior [31]; however, the lack of a control group precludes causal conclusions. In contrast, a recent RCT did not find that 6 months of aerobic exercise significantly improved aspects of memory and executive function relative to low-intensity balance and tone training [32]. In light of the promising evidence from previous pilot studies, from studies of older adults without chronic stroke [29], and from mechanistic animal studies [23 24], further research of exercise training among individuals with chronic stroke in large-scale, well-designed RCTs is needed.

Nevertheless, the physical ability of stroke survivors to participate in targeted exercise training is often limited. In fact, most stroke survivors adopt or return to sedentary lifestyles after rehabilitation [33]. Post-stroke physical deficits (e.g., balance) are associated with reduced activity participation [34]. Additional barriers to physical activity participation include low self-efficacy and social support [35]. Thus, other strategies to promote cognitive function need to be considered for this population.

An alternative or supplemental behavioral approach might be to intervene with cognitive and social enrichment activities to ameliorate cognitive impairment in the chronic stroke phase. The premise of this strategy is that by engaging in activities that stimulate higher-order cognition (e.g., memory and executive function), cognitive performance is improved and future cognitive decline is mitigated. Rodent models suggest that environment enrichment—e.g., housing in larger cages and in larger groups with varied environmental features—has various positive behavioural, neuro-anatomical, and molecular effects, including following stroke [36]. Few previous studies have tested this proposition among humans with chronic stroke. One 6-month pre-post study showed that a program that combined exercise training using the FAME program (2 days/week) with cognitive and social enrichment (1 day/week) was associated with benefits in aspects of memory and executive functions among individuals who had sustained a stroke at least 12 months prior [27]. Another pilot RCT compared this same multi-component intervention to a wait-list control and found benefits to aspects of executive functions, working memory, and physical functioning [37]. Whether these effects could be attributed primarily to the exercise training or to the social and cognitive activities could not be determined by this study design.

Thus, to better understand the relative effects of exercise training and of cognitive and social enrichment on cognitive function, we aim to conduct a 3-arm, parallel group proof-of-concept RCT comparing the following: 1) exercise training; 2) cognitive and social enrichment activities; and 3) an active control group consisting of stretching and toning activities. Each intervention arm will be 6 months in length and will be followed by a 6-month follow-up period. The results of this proof-of-concept RCT will be used to inform the design of a larger definitive trial. Specifically, it will confirm the feasibility of the study methods and procedures.

Methods and analysis

Design Outline

We will conduct a six-month proof-of-concept RCT and follow-up our study cohort for an additional six months (see Figure 1). There will be a dedicated research coordinator (unblinded) and trained assessor (blinded). Standardized protocols will be developed and study personnel will be trained by the research team. Assessments and intervention classes will occur at a research laboratory on the Vancouver General Hospital campus, Vancouver, Canada.

Recruitment

Recruitment advertisements will be placed in local community centers, stroke support groups, and newspapers in Greater Vancouver. Interested individuals will initially be screened by telephone by the research coordinator using both the inclusion criteria and the modified Physical Activity Readiness Questionnaire (PAR-Q) [38], a screening measure of physical readiness for exercise that requires clearance by a physician to engage in exercise. Those who appear eligible will be invited to an information session. During the information sessions, potential participants will be provided with details of the study and will have the opportunity to ask questions. A consent and screening session will be arranged for those who are interested in participating at the end of the information sessions. Those who remain eligible after the screening session will proceed to baseline assessments after their physician provides: 1) a written recommendation indicating their appropriateness to participate in an exercise program; and 2) a detailed description of their stroke (i.e., when it occurred, lesion location, and lesion type as defined by previous MRI or computed tomography scans).

Inclusion Criteria

Community-dwelling adults will be included who have had an ischemic or hemorrhagic stroke (confirmed by previous MRI or computed tomography scan). In addition, individuals must meet the following inclusion criteria: 1) are aged 55 years and over; 2) have a history of at least one single stroke of at least one year prior to study enrolment; 3) have a Mini-Mental State Examination (MMSE) [39] score of $\geq$ 20/30 at screening, including a perfect score on the 3-step command to ensure intact comprehension and ability to follow instructions; 4) are community-dwelling; 5) live in Greater Vancouver area; 6) able to comply with scheduled visits, treatment plan, and other trial procedures; 7) read, write, and speak English with acceptable visual and auditory acuity; 8) not expected to start or are stable on a fixed dose of cognitive medications (e.g., donepezil, galantamine, etc.) during the 12-month study period; 9) able to walk for a minimum of six metres with rest intervals with or without assistive devices; 10) based on interview, have an activity tolerance of 60 minutes with rest intervals; 11) not currently participating in any regular therapy or progressive exercise; and 12) provide a personally signed and dated informed consent document indicating that the individual (or a legally acceptable representative) has been informed of all pertinent aspects of the trial. In addition, an assent form will be provided at baseline and again at regular intervals.

Exclusion Criteria

Individuals will be excluded who are: 1) diagnosed with dementia of any type; 2) diagnosed with another type of neurodegenerative or neurological condition (e.g., Parkinson's disease) that affects cognitive function and mobility; 2) at high risk for cardiac complications during exercise or unable to self-regulate activity or to understand recommended activity level (i.e., Class C of the American Heart Risk Stratification Criteria); 3) have clinically important peripheral neuropathy or severe musculoskeletal or joint disease that impairs mobility, as determined by his/her family physician; 4) taking medications that may negatively affect cognitive function, such as anticholinergics, including agents with pronounced anticholinergic properties (e.g., amitriptyline), major tranquilizers (i.e., typical and atypical antipsychotics), and anticonvulsants (e.g., gabapentin, valproic acid, etc.); or 5) aphasia as judged by an inability to communicate by phone.

Measurement

There will be three primary measurement sessions: baseline, 6 months, and 12 months. Baseline measurements will be obtained prior to randomization. Additional secondary measures will be assessed monthly by unblinded assessors throughout the 12-month study. Data will be entered and scored using standard scoring procedures for each measure. Paper files will be held in secure filing cabinets and digital data will be stored on encrypted hard drives in laboratory areas with limited, key card access. All participant materials will be identified by identification number to maintain participant confidentiality.

Screening and Consent Session

For the screening and consent session, the study coordinator will re-administer the Physical Activity Readiness Questionnaire (PAR-Q) [38], a screening measure of physical readiness for exercise. Global cognitive function will be assessed using the MMSE [39] and the

MoCA [40]. Eligible participants will be provided a form to be completed by their family physician to confirm the inclusion/exclusion criteria.

Descriptors and Relevant Covariates

At baseline, general health, demographics, socioeconomic status, and education will be ascertained by a questionnaire. We will also document each participant's American Stroke Classification [41], medication history, and type (e.g., ischemic, haemorrhage), location (e.g., middle cerebral artery), and structure (e.g., posterior parietal cortex) of stroke from medical records/family physician. At each of the three primary measurement sessions, we will measure age in years, standing height in centimetres, and mass in kilograms. We will assess ADL using the self-report Functional Independence Measure [42]. Participants will complete the Functional Comorbidity Index to estimate the degree of comorbidity associated with physical functioning [43].

Primary Outcome Measure

Our primary measure of cognitive function will be the Alzheimer's Disease Assessment Scale-Cognitive-Plus (ADAS-Cog-Plus). The ADAS-Cog-Plus score is computed using a custom script [44] using the package 'mirt' in the statistical package R (www.r-project.org). The ADAS-Cog-Plus uses a multidimensional item response theory model to generate a global cognitive functioning score and standard error of measurement for that score from the items of the ADAS-Cog and other standard cognitive assessments. For the current study, we used the 13-item ADAS-Cog [45], Trail Making Test Parts A and B [46], Digit Span Forward and Backward [47], and Animal and Vegetable Fluency [47] as the input variables into the scoring algorithm. The scoring algorithm references data from the Alzheimer's Disease Neuroimaging Initiative sample, which was composed of approximately 50% MCI cases, 25% cognitively normal individuals, and 25% dementia cases. Lower scores represent better cognitive performance; specifically, ADAS-Cog-Plus scores of approximately -1.0 indicate healthy cognitive functioning, of 0.0 indicate MCI, and of 1.0 indicate dementia [44].

Secondary Outcome Measures

1) Executive Function

A computerized version of the Stroop task [48] will assess the response inhibition and selective attention components of executive function. The task will be completed usingthe program E-prime using a Windows-based computer and Cedrus RB-540 response pad. Color (e.g, RED, BLUE) and non-color (e.g., DISK, SCREEN) words will appear individually on the screen with 2000 ms duration and will be printed in one of three colors (blue, green or yellow). Participants are instructed to press the response pad button that is the same color as the font color of the word as quickly and accurately as possible. Following 18 practice trials, the task consists of 42 neutral trials (e.g., the word DISK printed in green font), 42 congruent trials (e.g., the word GREEN printed in green font), and 42 incongruent trials (e.g., the word GREEN printed in blue font) presented in random order. The outcome is the median response time for incongruent trials minus the median response time for congruent trials, using only trials with correct responses. Higher scores are indicative a stronger Stroop effect, and thus, poorer executive function.

2) Instrumental activities of daily living (IADLs)

IADLs will be assessed using the self-report Lawton and Brody [49] IADLs Scale. This scale subjectively assesses ability to telephone, shopping, food preparation, housekeeping,

laundry, mode of transportation, responsibility for own medication, and ability to handle finances.

3) General Balance and Mobility

We will use the Short Physical Performance Battery [50] to assess general mobility and balance. For the Short Physical Performance Battery, participants are assessed on performances of standing balance, walking, and sit-to-stand. Each component is rated out of four points, for a maximum of 12 points; a score < 9/12 predicts subsequent disability [50]. We will also measure knee extension (quadriceps) strength using the method employed by the physiological profile assessment [51] and grip strength (in kg) using a digital Jamar isometric hand dynamometer.

4) Mood

Depression is a prevalent clinical entity in stroke survivors – it has been reported to be as high as 38% [52] – and is negatively associated with cognitive function [53]. We will use the CES-D [54] to assess for depression, which asks participants to respond by indicating the frequency of 20 items. High scores indicate greater depressive symptoms.

5) Quality of Life

We will use the EQ-5D-3L [55] to assess health-related quality of life. The reliability and validity of the EQ-5D-3L in the stroke population have been established [56]. Participants indicate the number of problems within the following 5 domains: mobility, self-care, usual activities, pain and anxiety/depression. A health state utility value is calculated from the scores on each of the 5 domains. Lower scores indicate poorer health state. Scores lower than zero indicate a health state considered worse than death.

6) *Health Care Resource Utilization:* Participants will complete monthly health care resource use-diaries over the 6-month study period and use this information to respond to a health care resource utilization questionnaire administered at 3 and 6 months.

7) Objective Sleep Quality

We will use the MotionWatch 8© actigraphy system (MW8; camntech) a light weight, water-resistant, tri-axial wrist-worn accelerometer. The MW8 provides reliable, previously validated estimates of daytime activity and sleep quality including sleep duration (i.e., total time asleep), efficiency (i.e., actual sleep time expressed as a percentage of time in bed), and fragmentation (i.e., a measure of sleep disruption during the sleep window) [57] [58]. Participants will be fitted with the MW8 and provided detailed information on its features (i.e., the light sensor, event marker button, and status indicator). Participants will be instructed to press the event marker button each night when they started trying to sleep; and again each morning when they finished trying to sleep. Participants also will be given consensus sleep diary and asked to complete it upon awakening each morning. We will record sleep quality with the MW8 and sleep diary over 14 days.

8) Blood biomarkers

For those who decide to participate and consent to an Optional Blood Draw for Biomarkers Subject Information and Consent Form, a blood draw will be conducted at Vancouver General Hospital looking at changes in lipid profile and insulin sensitivity.

Monthly Measurement of Secondary Outcome Measures

1) Current Physical Activity Level

Current level of physical activity will be determined by the valid and reliable Community Health Activities Model Program for Seniors (CHAMPS) questionnaire [59]. This 41-item questionnaire assesses participation in various activities, including physical activities of different

intensities, for the previous 4 weeks. A metabolic equivalent (MET) is assigned to each activity. Participants will be asked to only report physical activity participation outside the research study.

2) Leisure Activity Level

Participation in leisure activity (e.g., hobbies, volunteering, etc.) will be determined by the Nottingham Leisure Questionnaire [60]. The validity and reliability of this questionnaire in the stroke population have been established [60]. Participants will be asked to only report leisure activity participation outside the research study.

Proof-of Concept Outcome Measures

Feasibility outcomes for delivering the intervention (i.e., adherence) will be measured throughout the 6-month intervention period. Class attendance will be recorded by the instructors.

Treatment Allocation and Concealment

After patients have signed informed consent to agree to be involved in the trial, they will be stratified into 2 groups by stroke status (1 versus ≥ 2 prior stroke events) and then randomly allocated with an allocation ratio of 2:2:3 (EX:Cog-Plus:CON, respectively) using permuted blocks (size intentionally withheld) within each stratum. For random number generation, each stratum will have its own seed using Minitab, a statistical package to generate uniform random integers, to create the allocation order within each block. The statistician (Dr. Goldsmith) will hold the randomization book and will give out the allocations of individual patients one-at-a-time to the 3 groups, and so these allocations will be concealed from patients, all study personnel and the investigators, except Dr. Goldsmith, until the interventions are implemented. The specific blocks will be revealed for use in the statistical analyses once the database has been cleaned and is ready for the statistical analyses. This process will allow the blocking restriction to be considered in the data analyses along with the integrity of the randomization.

Interventions

All exercise-based classes will be led by instructors who have formal expertise in delivering group exercise programs to older adults. All classes will be 60 minutes in duration. All classes will have a maximum participant to instructor ratio of 4:1. Class attendance will be recorded by the instructors. To minimize contamination, only one class will occur at any given time in the same facility. In addition, there will be a minimum of 30 minutes between classes at any given facility. All intervention groups will include twice-weekly classes of 60 minutes each over 26 weeks. Fidelity across instructors and across time will be ensured by providing instructors with detailed protocols including pictures; regular observation and intervention classes by study PI and coordinator and auditing with standard checklist to ensure intervention content is delivered accurately and consistently; and videotaping classes from each intervention arm across time.

EX Group

The EX program is a multi-component intervention based in part on the FAME program [61]. We have developed specific guidelines and increments for each exercise in this program to provide safe and objective progression of the participants. Participants will be familiarized with the Borg Rating of Perceived Exertion (RPE) and the scale will be visible in the room. We have previously used the RPE in individuals with chronic stroke and found it representative of myocardial exertion [62]. Each class will have a 10-minute warm-up, three core components—

strength training (20 minutes); aerobic/agility training (20 minutes); and balance training (5 minutes)—and will end with a 5-minute cool down. Strength training will consist of calf raises, squats, bicep curls, tricep extensions, and an alternating fifth activity of either sliding back lunges or standing leg abduction. Exercises will be progressed by adding weight (e.g., dumbbells, completing single calf raise versus double calf raise) or altering movement tempo (e.g., fast concentric motion followed by slow eccentric motion, adding hold at bottom of squat). Aerobic/agility exercises will include heal and toe tapping, low and high knee marches, stepper exercise, agility ladders, and figure 8 walking. Initially, participants will be asked to complete the exercise at an intensity corresponding to a RPE of 12. Exercise intensity will be progressed at a rate of approximately 1 RPE/month, with a final target RPE of 16 during month 6. Balance exercises will have participants complete various movements (e.g., hit balloons, throw ball against wall, walk forward and backward, close eyes, rotate trunk, move arms) while standing and with feet in either side-by-side, semi-tandem, or fully-tandem positions. Heart rate monitors (Polar RS400) will be worn throughout class, with measurement occurring before class, at least twice during class, and at the end of class. The BORG RPE will be administered at least twice during class and at the end of class. Exercise difficulty (e.g., added weight, stepper height) will be recorded during each class, and the Timed-Up-and-Go task and Short Physical Performance Battery will be completed monthly to provide objective performance tracking.

Cog-Plus Group

We have designed this program based on the feedback received from our pilot study [27] and based on current evidence [63]. In addition, we aimed to design a program that could feasibly be implemented in the community with minimal resources. Because impairment in multiple cognitive domains is common following stroke [8-10], the cognitive exercises included in the program targeted various aspects of cognition, including learning and memory, processing speed, attention, working memory, and executive functions. Each class will begin with the participants being asked to memorize a 7-item word list. Next, each participant will complete approximately 15 minutes of the brain training program Lumosity using an individually-issued Apple iPad. Lumosity consists of various short games (typically 1 to 5 minutes) that target various aspects of cognition (e.g., working memory, divided attention, processing speed). Each class, participants will be encouraged to complete at least 5 distinct games. For the remaining class time (approximately 30 minutes), the participants will complete a variety of social games and mental activities in pairs or as entire class. Some of these activities will utilize apps on the Apple iPads (e.g., *Heads-Up*, *Teledoodle*), and others are based on improvisation and mental activities from the PERK program [64]. At the end of class, participants will recall as many of the words from the word list; they will use the Notes program on the iPad to record the recalled words. Approximately every month, the class instructor will meet individually with the participants to show performance progress on the Lumosity training program and to discuss outstanding concerns and areas of improvement (e.g., short-term memory, speed of responding). Every month, the 7-item word list will be replaced with a 15-item word list, and participants will be requested to recall those words immediately, as well as at the end of the class.

CON Group

The CON program will follow the protocol used in Dr. Liu-Ambrose's previous RCT [65]. The CON protocol will consist of stretches, deep breathing and relaxation techniques, general posture education, general core control exercises, grip strength and dexterity exercises, and light

isometric toning exercises. Some exercises from the EX program will also be included but in a simplified format without progression (e.g., double calf raises, heal and toe tapping, balance exercises). Once a month, the class will consist of educational lecture and will include topics such as sleep hygiene, goal setting, and nutrition. This group will serve to control for confounding variables such as physical training received by traveling to the community centre for twice-weekly classes and changes in lifestyle secondary to study participation. The Timed-Up-and-Go task[66] will be completed on a monthly basis to allow for objective physical performance tracking.

Data and Adverse Events Monitoring

A Data and Safety Monitoring Committee will be established by co-investigators who will be independent from the day-to-day conduct of the study and from the study funders. Drs. Hsiung, Davis, Middleton, and Goldsmith will review all adverse events reported in the study on a monthly basis. They will stop the study if the adverse events data demonstrate any hazards that are the result of the intervention. They will also ensure data sharing and fidelity. Data provided to project team members will exclude identifying participant information.

Strategies to Promote Adherence

We will implement strategies to promote adherence during the 6-month intervention as recommended by the literature [67-71]. These will include: 1) monthly phone calls by the unblinded research coordinators to encourage adherence to classes; multiple contacts have been shown to be more effective than single exposures [67]; 2) discussing participant barriers and developing coping plans and action plans [68]; 3) setting implementation intentions and concrete plans [69]; and 4) encourage participants to continually self-monitor their progress with monthly calendars provided by the study. This strategy has been identified as the most successful behavioural/cognitive approach when compared to all other current adherence techniques [70].

Sample Size Calculation

We have designed our trial to allow the evaluation of statistical significance of the treatment effect between groups on the ADAS-Cog-Plus. A number of pharmaceutical RCTs in vascular dementia [72-75] – a population highly relevant to our proposed study – have shown positive cognitive effects as measured by the ADAS-Cog, and it has been suggested that the ADAS-Cog-Plus shows greater sensitivity to underlying changes in cognition [44]. A previous RCT of physical activity in older adults at risk for Alzheimer's disease with ADAS-Cog as the primary outcome measure demonstrated a standardized effect size of 0.60 [17]. We used interim results from our PROMOTE study [76] – an exercise RCT in adults with mild sub-cortical ischaemic vascular cognitive impairment – for our sample size estimation. Based on data collected from 15 participants who have completed the RCT, we found the mean change in the ADAS-Cog score was 2.7 (SD=2.3) and 0.87 (SD=3.4) for the exercise training group and the control group, respectively. The minimally clinically relevant change (MCRC) on the ADAS-Cog varies between 3 and 5 points, with a change of $\geq$ 4 being recommended by the Food and Drug Administration [77]. Recently, Schrag and colleagues [78] established the MCRC empirically using data collected from the Alzheimer's disease Neuroimaging Initiative. They found that a 3-point change on the ADAS-Cog is an appropriate MCRC. Assuming a mean change of 3 points on the ADAS-Cog for both the EX and Cog-Plus groups and a mean change of 1 point for the CON group at 6 months, a common standard deviation of 2.85, and an alpha of 0.05, 39

participants per group (i.e., total sample of 117) will provide a power greater than 0.80 [79]. We then assumed a standardized effect size of 0.7 for the ADAS-Cog-Plus, as it has greater sensitivity to changes in cognitive function compared with the 11- or 13-item ADAS-Cog. After accounting for 15% attrition and 2:2:3 allocation, 34 participants were randomized to the EX group, 34 to the ENRICH group, and 52 to the BAT group, for a total sample of 120.

Statistical Analyses

Primary Outcome

This analysis will follow the intention-to-treat principal, such that all randomized participants will be included to estimate treatment effects, irrespective of deviations from treatment protocol (e.g., loss to follow-up, non-compliance). This will be done using linear mixed models using maximum likelihood estimation. The model will include random intercepts and slopes, and fixed effects of time (baseline, month 6, month 12), treatment assignment (CON, EX, Cog-Plus), and their interaction. Baseline MMSE and ADAS-Cog-Plus scores will also be included as fixed effect covariates. Time will be specified as a categorical variable, thus allowing us to examine treatment differences at the primary endpoint (month 6) and then, as a secondary objective, whether those differences persist at the 6-month follow-up (month 12). Two planned simple contrasts will be performed using the Dunnett test [80]. These contrasts will be employed to assess differences between: 1) the EX group and the CON group; and 2) the Cog-Plus group and the CON group. The overall alpha will be set at 0.05. A secondary complete-case analysis will be conducted using this linear mixed model, in which participants with valid data at all time points will be included. As an exploratory strategy, multiple imputation will be used to judge the impact of missingness on the conclusions drawn from this study [81].

Secondary & Tertiary Outcomes

Analyses will be descriptive; no alpha has been allocated. Point and interval estimates for the effect of the intervention on each of the secondary outcomes at six and 12 months will be determined separately using linear mixed models. Multiple linear regression analyses will also be performed to explore the association between change in cognitive function, after accounting for experimental group, baseline age, baseline global cognition, and: 1) superior treatment adherence; 2) change in physical activity levels outside the research protocol; and 3) change in general balance and mobility. Randomization integrity will be determined by examining bias in the blocking sequence used to produce allocation sequence.

Economic Evaluation – A Cost-utility analysis

Our economic evaluation will examine the incremental costs and effects generated by using a 1) 6-month targeted exercise training program or a 2) 6-month cognitive and social enrichment program among older adults with chronic stroke compared with a 6-month stretch and tone program (i.e., control; comparator). The outcome of our cost effectiveness analysis is the incremental cost-utility ratio (ICUR). By definition, an ICUR is the difference between the mean costs of providing the competing intervention divided by the incremental difference in QALYs, where ICUR=Δ Cost/Δ QALY [82,83]. QALYs are calculated based on the quality of life of a patient (measured using health state utility values estimated from the EQ-5D-3L) in a given health state and the time spent in that health state. For any missing data, we will use a combination of imputation and bootstrapping to quantify uncertainty due to missing values [84,85].

Proof-of-Concept Outcomes

Feasibility outcomes – such as recruitment rate, withdrawal rate, adherence, and number of adverse events – will be treated as binary, with "success" indicating the protocol is sufficiently robust to move forward with the large RCT with only small or no adaptation required, and "revise" indicating a need for more substantive change before proceeding [86].

References

1. Seshadri S, Beiser A, Kelly-Hayes M, et al. The lifetime risk of stroke: estimates from the Framingham Study. *Stroke* 2006;37(2):345-50.
2. Rockwood K, Wentzel C, Hachinski V, et al. Prevalence and outcomes of vascular cognitive impairment. Vascular Cognitive Impairment Investigators of the Canadian Study of Health and Aging. *Neurology* 2000;54(2):447-51.
3. Desmond DW, Erkinjuntti T, Sano M, et al. The cognitive syndrome of vascular dementia: implications for clinical trials. *Alzheimer Dis Assoc Disord* 1999;13 Suppl 3:S21-9.
4. Erkinjuntti T, Bowler JV, DeCarli CS, et al. Imaging of static brain lesions in vascular dementia: implications for clinical trials. *Alzheimer Dis Assoc Disord* 1999;13 Suppl 3:S81-90.
5. Pantoni L, Leys D, Fazekas F, et al. Role of white matter lesions in cognitive impairment of vascular origin. *Alzheimer Dis Assoc Disord* 1999;13 Suppl 3:S49-54.
6. Fratiglioni L, De Ronchi D, Aguero-Torres H. Worldwide prevalence and incidence of dementia. *Drugs & aging* 1999;15(5):365-75.
7. Kokmen E, Whisnant JP, O'Fallon WM, et al. Dementia after ischemic stroke: a population-based study in Rochester, Minnesota (1960-1984). *Neurology* 1996;46(1):154-9.
8. Zinn S, Bosworth HB, Hoenig HM, et al. Executive function deficits in acute stroke. *Arch Phys Med Rehabil* 2007;88(2):173-80.
9. Tatemichi TK, Desmond DW, Stern Y, et al. Cognitive impairment after stroke: Frequency, patterns, and relationship to functional abilities. *Journal of Neurology, Neurosurgery, & Psychiatry* 1994;57:202-07.
10. Sun JH, Tan L, Yu JT. Post-stroke cognitive impairment: epidemiology, mechanisms and management. *Ann Transl Med* 2014;2(8):80.
11. Pasquini M, Leys D, Rousseaux M, et al. Influence of cognitive impairment on the institutionalisation rate 3 years after a stroke. *Journal of neurology, neurosurgery, and psychiatry* 2007;78(1):56-9.
12. Edwards JD, Koehoorn M, Boyd LA, et al. Is health-related quality of life improving after stroke? A comparison of health utilities indices among Canadians with stroke between 1996 and 2005. *Stroke; a journal of cerebral circulation*;41(5):996-1000.
13. Patel MD, Coshall C, Rudd AG, et al. Cognitive impairment after stroke: clinical determinants and its associations with long-term stroke outcomes. *J Am Geriatr Soc* 2002;50(4):700-6.
14. Heyn P, Abreu BC, Ottenbacher KJ. The effects of exercise training on elderly persons with cognitive impairment and dementia: a meta-analysis. *Archives of physical medicine and rehabilitation* 2004;85(10):1694-704.
15. Colcombe S, Kramer AF. Fitness effects on the cognitive function of older adults: a meta-analytic study. *Psychol Sci* 2003;14(2):125-30.
16. Etnier JL, Nowell PM, Landers DM, et al. A meta-regression to examine the relationship between aerobic fitness and cognitive performance. *Brain research reviews* 2006;52(1):119.
17. Lautenschlager NT, Cox KL, Flicker L, et al. Effect of Physical Activity on Cognitive Function in Older Adults at Risk for Alzheimer Disease: A Randomized Trial. *Jama* 2008;300(9):1027-37.
18. Baker LD, Frank LL, Foster-Schubert K, et al. Effects of aerobic exercise on mild cognitive impairment: a controlled trial. *Archives of neurology* 2010;67(1):71-9.

19. Liu-Ambrose T, Nagamatsu LS, Graf P, et al. Resistance training and executive functions: a 12-month randomized controlled trial. *Archives of internal medicine* 2010;170(2):170-8.
20. Liu-Ambrose T, Nagamatsu LS, Voss MW, et al. Resistance training and functional plasticity of the aging brain: A 12-month randomized controlled trial. *Neurobiology of Aging* 2012;33:1690-98.
21. Voss MW, Vivar C, Kramer AF, et al. Bridging animal and human models of exercise-induced brain plasticity. *Trends Cogn Sci* 2013;17:525-44.
22. Cotman CW, Berchtold NC, Christie L-A. Exercise builds brain health: key roles of growth factor cascades and inflammation. *Trends in Neurosciences* 2007;30:464-72.
23. Mason JL, Ye P, Suzuki K, et al. Insulin-like growth factor-1 inhibits mature ologodentrocyte apoptosis during primary demyelination. *The Journal of Neuroscience* 2000;20:5703-08.
24. Ye P, Carson JA, D'Ercole AJ. In vivo actions of insulin-like growth factor-1 (IGF-1) on brain myelination: Studies of IGF-1 and IGF binding protein-1 (IGFBP-1) transgenic mice. *The Journal of Neuroscience* 1995;15:7344-56.
25. Cumming TB, Tyedin K, Churilov L, et al. The effect of physical activity on cognitive function after stroke: a systematic review. *Int Psychogeriatr* 2012;24(4):557-67.
26. McDonnell MN, Smith AE, Mackintosh SF. Aerobic exercise to improve cognitive function in adults with neurological disorders: a systematic review. *Archives of physical medicine and rehabilitation*;92(7):1044-52.
27. Rand D, Eng JJ, Liu-Ambrose T, et al. Feasibility of a 6-month exercise and recreation program to improve executive functioning and memory in individuals with chronic stroke. *Neurorehabil Neural Repair* 2010;24(8):722-9.
28. Cumming TB, Tyedin K, Churilov L, et al. The effect of physical activity on cognitive function after stroke: a systematic review. *International Psychogeriatrics*;FirstView:1-11.
29. Quaney BM, Boyd LA, McDowd JM, et al. Aerobic exercise improves cognition and motor function poststroke. *Neurorehabil Neural Repair* 2009;23(9):879-85.
30. Moore SA, Hallsworth K, Jakovljevic DG, et al. Effects of Community Exercise Therapy on Metabolic, Brain, Physical, and Cognitive Function Following Stroke: A Randomized Controlled Pilot Trial. *Neurorehabil Neural Repair* 2015;29(7):623-35.
31. Marzolini S, Oh P, McIlroy W, et al. The effects of an aerobic and resistance exercise training program on cognition following stroke. *Neurorehabil Neural Repair* 2013;27(5):392-402.
32. Tang A, Eng JJ, Krassioukov AV, et al. High- and low-intensity exercise do not improve cognitive function after stroke: A randomized controlled trial. *J Rehabil Med* 2016;48(10):841-46.
33. Lofgren B, Nyberg L, Mattsson M, et al. Three years after in-patient stroke rehabilitation: A follow-up study. *Cerebrovascular diseases (Basel, Switzerland)* 1999;9(3):163-70.
34. Robinson CA, Shumway-Cook A, Matsuda PN, et al. Understanding physical factors associated with participation in community ambulation following stroke. *Disability and rehabilitation*;33(12):1033-42.
35. Morris J, Oliver T, Kroll T, et al. The importance of psychological and social factors in influencing the uptake and maintenance of physical activity after stroke: a structured review of the empirical literature. *Stroke research and treatment*;2012:195249.
36. van Praag H, Kempermann G, Gage FH. Neural consequences of environmental enrichment. *Nature Reviews Neuroscience* 2000;1:191-98.

37. Liu-Ambrose T, Eng JJ. Exercise training and recreational activities to promote executive functions in chronic stroke: a proof-of-concept study. *J Stroke Cerebrovasc Dis* 2015;24(1):130-7.
38. Canadian Society for Exercise Physiology. Par-Q and You. Gloucester, Ontario, Canada: Canadian Society of Exercise Physiology, 1994:1-2.
39. Folstein MF, Folstein SE, McHugh PR. "Mini-mental state". A practical method for grading the cognitive state of patients for the clinician. *J Psychiatr Res* 1975;12(3):189-98.
40. Nasreddine ZS, Phillips NA, Bedirian V, et al. The Montreal Cognitive Assessment, MoCA: a brief screening tool for mild cognitive impairment. *J Am Geriatr Soc* 2005;53(4):695-9.
41. Kelly-Hayes M, Robertson JT, Broderick JP, et al. The American Heart Association Stroke Outcome Classification: executive summary. *Circulation* 1998;97(24):2474-8.
42. Granger CV. The emerging science of functional assessment: our tool for outcomes analysis. *Arch Phys Med Rehabil* 1998;79(3):235-40.
43. Groll DL, To T, Bombardier C, et al. The development of a comorbidity index with physical function as the outcome. *J Clin Epidemiol* 2005;58(6):595-602.
44. Mungas D, Crane P, Dowling M, et al. Optimizing cognitive outcome measures in AD clinical trials: Technical Summary, 2013.
45. Rosen WG, Mohs RC, Davis KL. A new rating scale for Alzheimer's disease. *The American Journal of Psychiatry* 1984;141(11):1356-64.
46. Spreen O, Strauss E. A Compendium of Neurological Tests. 2nd ed. New York: Oxford University Press, Inc. 1998.
47. Wechsler D. Wechsler Adult Intelligence Scale - Revised. New York, New York: The Psychological Corporation, Harcourt Brace Jovanovich., 1981.
48. Trenerry M, Crosson B, DeBoe J, et al. Stroop Neuropsychological Screening Test: Psychological Assessment Resources, 1988.
49. Lawton MP, Brody EM. Assessment of older people: self-maintaining and instrumental activities of daily living. *Gerontologist* 1969;9(3):179-86.
50. Guralnik JM, Ferrucci L, Simonsick EM, et al. Lower-extremity function in persons over the age of 70 years as a predictor of subsequent disability. *New Engl J Med* 1995;332:556-61.
51. Lord SR, Menz HB, Tiedemann A. A physiologic profile approach to falls risk assessment and prevention. *Physical Therapy* 2003;83:237-52.
52. Carod-Artal J, Egido JA, Gonzalez JL, et al. Quality of life among stroke survivors evaluated 1 year after stroke: experience of a stroke unit. *Stroke* 2000;31(12):2995-3000.
53. Berger AK, Fratiglioni L, Winblad B, et al. Alzheimer's disease and depression: preclinical comorbidity effects on cognitive functioning. *Cortex* 2005;41(4):603-12.
54. Radloff LS. The CES-D Scale: A Self-Report Depression Scale for Research in the General Population. *Applied Psychological Measurement* 1977;1(3):385-401.
55. Ware J, Jr., Kosinski M, Keller SD. A 12-Item Short-Form Health Survey: construction of scales and preliminary tests of reliability and validity. *Med Care* 1996;34(3):220-33.
56. Bohannon RW, Maljanian R, Lee N, et al. Measurement properties of the short form (SF)-12 applied to patients with stroke. *Int J Rehabil Res* 2004;27(2):151-4.
57. Validation of a new actigraph motion watch versus polysomnography on 70 healthy and suspected sleep-disordered subjects. JOURNAL OF SLEEP RESEARCH; 2012. WILEY-BLACKWELL 111 RIVER ST, HOBOKEN 07030-5774, NJ USA.

58. Middleton B, Hampton S. Comparison of new activity monitors (MW8) and PRO-Diary Motion (PDM) containing accelerometer with existing activity monitors (AWL): University of Surrey, 2012:1-38.
59. Stewart AL, Mills KM, King AC, et al. CHAMPS physical activity questionnaire for older adults: Outcomes for interventions. *Medicine & Science in Sports & Exercise* 2001;33(7):1126-41.
60. Drummond AE, Parker CJ, Gladman JR, et al. Development and validation of the Nottingham Leisure Questionnaire (NLQ). *Clin Rehabil* 2001;15(6):647-56.
61. Pang MY, Eng JJ, Dawson AS, et al. A community-based fitness and mobility exercise program for older adults with chronic stroke: a randomized, controlled trial. *J Am Geriatr Soc* 2005;53(10):1667-74.
62. Eng JJ, Chu KS, Dawson AS, et al. Functional walk tests in individuals with stroke: relation to perceived exertion and myocardial exertion. *Stroke* 2002;33(3):756-61.
63. Basak C, Boot WR, Voss MW, et al. Can training in a real-time strategy video game attenuate cognitive decline in older adults? *Psychol Aging* 2008;23(4):765-77.
64. Moritz R, Neeson I, Latif F, et al. Perk Activities: The Social Experience that Stimulates 2017 [Available from: https://www.perkactivities.com/ accessed December 6, 2017 2017.
65. Liu-Ambrose T, Khan KM, Eng JJ, et al. Resistance and agility training reduce fall risk in women aged 75 to 85 with low bone mass: a 6-month randomized, controlled trial. *Journal of the American Geriatrics Society* 2004;52(5):657-65.
66. Shumway-Cook A, Brauer S, Woollacott M. Predicting the probability for falls in community-dwelling older adults using the Timed Up & Go Test. *Phys Ther* 2000;80(9):896-903.
67. Hillsdon M, Foster C, Thorogood M. Interventions for promoting physical activity. *Cochrane Database Syst Rev* 2005(1):CD003180.
68. Sniehotta FF, Scholz U, Schwarzer R. Action plans and coping plans for physical exercise: A longitudinal intervention study in cardiac rehabilitation. *Br J Health Psychol* 2006;11(Pt 1):23-37.
69. Gollwitzer PM, Sheeran P. Implementation intentions and goal achievement: A meta-analysis of effects and processes. *Advances in Experimental Social Psychology* 2006;38:69-119.
70. Michie S, Abraham C, Whittington C, et al. Effective techniques in healthy eating and physical activity interventions: a meta-regression. *Health Psychol* 2009;28(6):690-701.
71. Hillsdon M, Foster C, Cavill N, et al. The effectiveness of public health interventions for increasing physical activity among adults: a review of reviews. *London: Health Development Agency* 2005:1-40.
72. Orgogozo JM, Rigaud AS, Stoffler A, et al. Efficacy and safety of memantine in patients with mild to moderate vascular dementia: a randomized, placebo-controlled trial (MMM 300). *Stroke* 2002;33(7):1834-9.
73. Pratt RD, Perdomo CA. Donepezil-treated patients with probable vascular dementia demonstrate cognitive benefits. *Ann N Y Acad Sci* 2002;977:513-22.
74. Wilcock G, Mobius HJ, Stoffler A. A double-blind, placebo-controlled multicentre study of memantine in mild to moderate vascular dementia (MMM500). *Int Clin Psychopharmacol* 2002;17(6):297-305.
75. Wilkinson D, Doody R, Helme R, et al. Donepezil in vascular dementia: a randomized, placebo-controlled study. *Neurology* 2003;61(4):479-86.

76. Liu-Ambrose T, Eng JJ, Boyd LA, et al. Promotion of the mind through exercise (PROMoTE): a proof-of-concept randomized controlled trial of aerobic exercise training in older adults with vascular cognitive impairment. *BMC Neurol*;10:14.
77. Food and Drug Administration. Peripheral and Central Nervous System Drugs Advisory Committee Meeting. Rockville, MD: Department of Health and Human Services, Public Health Service, 1989:227.
78. Schrag A, Schott JM. What is the clinically relevant change on the ADAS-Cog? *J Neurol Neurosurg Psychiatry*;83(2):171-3.
79. Portney LG, Watkins MP. Foundations of Clinical Research: Applications to Practice. Norwalk: Appleton and Lange 1993.
80. Dunnett C, Goldsmith C. When and how to decide to do multiple comparisons. In: Buncher CR, Tsay J-Y, eds. Statistics in the Pharmaceutical Company. 3rd ed. Boca Raton, FL: CRC Press, Taylor and Francis Group 2006:421-51.
81. Little RJ, D'Agostino R, Cohen ML, et al. The prevention and treatment of missing data in clinical trials. *N Engl J Med* 2012;367(14):1355-60.
82. Drummond MF, Sculpher MJ, Torrance GW, et al. Methods for the Economic Evaluation for Health Car Programmes. 3rd ed. New York, New York: Oxford University Press 2005.
83. Glick HA, Doshi JA, Sonnad SA, et al. Economic Evaluation in Clinical Trials. 1st ed. New York, New York: Oxford University Press 2007.
84. Briggs AH, Gray AM. Handling uncertainty when performing economic evaluation of healthcare interventions. *Health Technology Assessment* 1999;3:1-134.
85. Oostenbrink JB, Al MJ, Rutten-van Molken MP. Methods to analyse cost data of patients who withdraw in a clinical trial setting. *PharmacoEconomics* 2003;21:1103-12.
86. Thabane L, Ma J, Chu R, et al. A tutorial on pilot studies: the what, why and how. *BMC medical research methodology*;10(1):1.
