# Supplementary material for: Effect of Exercise Training or Complex Mental and Social Activities on Cognitive Function in Adults With Chronic Stroke: A Randomized Clinical Trial
Source: JAMA Netw Open. 2022 Oct 13;5(10):e2236510. doi: 10.1001/jamanetworkopen.2022.36510 (PMC9561961; doi:10.1001/jamanetworkopen.2022.36510)
Supplement: Supplement 2. — eTable 1. Estimated Between-Group Difference at Month 6 and Month 12 for Complete Case Analyses eTable 2. Estimated Marginal Means for Primary and Secondary Outcome Variables by Treatment Group [file jamanetwopen-e2236510-s002.pdf]

Supplemental Online Content

Liu-Ambrose T, Falck RS, Dao E, et al. Effect of exercise training or complex mental and social activities on cognitive function in adults with chronic stroke: a randomized clinical trial. *JAMA Netw Open*. 2022;5(10):e2236510. doi:10.1001/jamanetworkopen.2022.36510

eTable 1. Estimated Between-Group Difference at Month 6 and Month 12 for Complete Case Analyses

eTable 2. Estimated Marginal Means for Primary and Secondary Outcome Variables by Treatment Group

This supplemental material has been provided by the authors to give readers additional information about their work.

© 2022 Liu-Ambrose T et al. *JAMA Network Open.*

eTable 1: Estimated Between Group Difference at Month 6 and Month 12 for Complete Case Analyses

	Adjusted between group difference (EX - BAT)		Adjusted between group difference (ENRICH - BAT)	
**Variable**	**Month 6 (95% CI)**	**Month 12 (95% CI)**	**Month 6 (95% CI)**	**Month 12 (95% CI)**
**Primary Outcome**				
Alzheimer's Disease Assessment Scale-Cognitive-Plus	-0.26 (-0.46, -0.06)	-0.08 (-0.28, 0.12)	-0.12 (-0.31, 0.07)	-0.08 (-0.28, 0.11)
**Secondary Outcomes**				
13-item Alzheimer's Disease Assessment Scale-Cognitive (0-90 [worst])	-2.39 (-4.44, -0.35)	-0.12 (-2.17, 1.93)	-0.93 (-2.88, 1.03)	0.11 (-1.84, 2.06)
Stroop Interference Ratio[a]	-0.01 (-0.05, 0.04)	-0.02 (-0.06, 0.03)	-0.03 (-0.07, 0.02)	-0.01 (-0.06, 0.03)
Instrumental Activities of Daily Living (0-8 [best])	-0.24 (-3.14, 2.66)	-0.18 (-3.09, 2.74)	-0.29 (-3.18, 2.60)	-0.10 (-3.01, 2.80)
Short Physical Performance Battery (0-12 [best])	-0.35 (-3.14, 2.44)	0.27 (-2.52, 3.06)	-0.71 (-3.50, 2.08)	-0.19 (-2.98, 2.59)
Gait Speed (m/s)	-0.01 (-0.10, 0.08)	-0.01 (-0.10, 0.09)	-0.04 (-0.13, 0.04)	-0.01 (-0.09, 0.07)
Six Minute Walk Test (meters)	3.18 (-23.00, 29.31)	0.21 (-25.80, 26.18)	-21.83 (-46.10, 2.46)	-10.38 (-34.80, 14.01)
Physical Activity (kcal/week)[b]	-919 (-1873, 35)	-1040 (-2034, -46)	-677 (-1560, 205)	-695 (-1630, 240)

[a] Stroop Interference Ratio is calculated as: Incongruent Median Reaction Time (ms) - Congruent Median Reaction Time (ms) / Congruent Median Reaction Time (ms).
[b] Measured using the Community Health Model Activities Program (CHAMPS) physical activity questionnaire.

© 2022 Liu-Ambrose T et al. *JAMA Network Open.*

eTable 2. Estimated Marginal Means for Primary and Secondary Outcome Variables by Treatment Group

	EX (n=34)			ENRICH (n=34)			BAT (n=52)		
**Variable**[ǂ]	**Baseline**	**Month 6**	**Month 12**	**Baseline**	**Month 6**	**Month 12**	**Baseline**	**Month 6**	**Month 12**
**Primary Outcome**									
Alzheimer's Disease Assessment Scale-Cognitive-Plus[a]	0.39 (0.77)	-0.32 (0.08)	-0.22 (0.08)	0.12 (0.71)	-0.19 (0.08)	-0.21 (0.08)	0.16 (0.88)	-0.08 (0.06)	-0.13 (0.06)
**Secondary Outcomes**									
13-item Alzheimer's Disease Assessment Scale-Cognitive (0-90)[b]	18.16 (7.47)	12.51 (0.76)	12.97 (0.79)	16.42 (6.29)	13.99 (0.76)	13.44 (0.77)	17.19 (8.00)	14.95 (0.61)	13.35 (0.62)
Stroop Interference Ratio[c]	0.16 (0.11)	0.15 (0.02)	0.14 (0.02)	0.16 (0.12)	0.13 (0.02)	0.15 (0.02)	0.16 (0.15)	0.15 (0.01)	0.16 (0.01)
Instrumental Activities of Daily Living (0-8)[d]	6.82 (1.83)	7.47 (1.05)	7.57 (1.05)	7.00 (1.26)	7.42 (1.04)	7.64 (1.05)	6.79 (1.73)	7.71 (1.05)	7.74 (1.05)
Short Physical Performance Battery (0-12)[e]	7.44 (2.52)	9.49 (1.01)	9.36 (1.01)	8.15 (2.48)	9.13 (1.01)	8.90 (1.01)	8.69 (2.81)	9.84 (1.01)	9.10 (1.01)
Gait Speed, m/s	0.76 (0.32)	0.92 (0.03)	0.88 (0.03)	0.88 (0.35)	0.92 (0.03)	0.89 (0.03)	0.91 (0.31)	0.96 (0.03)	0.90 (0.03)
Six-Minute Walk Test, m	291.09 (147.83)	359.81 (9.87)	341.51 (10.13)	342.47 (143.44)	342.62 (9.70)	336.12 (9.79)	340.79 (131.85)	368.32 (8.03)	349.43 (8.19)
Physical Activity (kcal/wk)[f]	3262 (2500)	2893 (343)	2666 (353)	3535 (2718)	3236 (326)	3047 (348)	3536 (2255)	3928 (276)	3793 (295)

ǂ Mean and standard deviation reported for baseline; estimated marginal means and standard error reported for month 6 and month 12.

a Lower Alzheimer's Disease Assessment Scale-Cognitive-Plus scores indicate better cognitive performance. The range of ADAS-Cog-Plus scores is from -3.46 to 4.31. Scores approximately of -1.0 indicate healthy cognitive functioning, of 0.0 indicate mild cognitive impairment, and of 1.0 indicate dementia.

b Lower 13-item Alzheimer's Disease Assessment Scale-Cognitive scores indicate better cognitive performance.

c Stroop Interference Ratio is calculated as: Incongruent Median Reaction Time (ms) - Congruent Median Reaction Time (ms) / Congruent Median Reaction Time (ms); lower scores indicate less interference, or better performance.

d The (Lawton) Instrumental Activities of Daily Living ranges from 0 (worst, dependent) to 8 (best, independent). A score of 7 would indicate someone who is largely independent but can't manage finances or perform housekeeping tasks.

e The Short Physical Performance Battery ranges from 0 (worst) to 12 (best); scores ≤ 9 indicate increased risk for disability.

f Measured using the Community Health Model Activities Program (CHAMPS) physical activity questionnaire.

© 2022 Liu-Ambrose T et al. *JAMA Network Open.*
