# Supplementary material for: Effect of Exercise Training or Complex Mental and Social Activities on Cognitive Function in Adults With Chronic Stroke: A Randomized Clinical Trial
Source: JAMA Netw Open. 2022 Oct 13;5(10):e2236510. doi: 10.1001/jamanetworkopen.2022.36510 (PMC9561961; doi:10.1001/jamanetworkopen.2022.36510)
Supplement: Supplement 3. — Data Sharing Statement [file jamanetwopen-e2236510-s003.pdf]

Data Sharing Statement

Liu-Ambrose. Effect of Exercise Training or Complex Mental and Social Activities on Cognitive Function in Adults With Chronic Stroke. *JAMA Netw Open*. Published October 13, 2022. doi:10.1001/jamanetworkopen.2022.36510

Data
Data available: No

Additional Information
Explanation for why data not available: Our original ethics and consent did not state it would be shared with others. However, de-identified participant data will be made available by the corresponding author to others who propose a reasonable scientific request and obtain appropriate ethics.
